# Seizure Clusters: Current Concepts in Definition and Treatment

**DOI:** 10.3390/jcm15051847

**Published:** 2026-02-28

**Authors:** Gemma Bassani, Elena Pasini, Barbara Mostacci, Lidia Di Vito, Lorenzo Ferri, Lorenzo Muccioli, Francesca Bisulli

**Affiliations:** 1 Department of Biomedical and NeuroMotor Sciences (DIBINEM), University of Bologna, 40126 Bologna, Italy; gemma.bassani@studio.unibo.it; 2 IRCCS Istituto delle Scienze Neurologiche di Bologna, Full Member of the ERN EpiCARE, 40139 Bologna, Italy; barbara.mostacci@isnb.it (B.M.); lidia.divito@ausl.bologna.it (L.D.V.); lorenzo.ferri@ausl.bologna.it (L.F.); lorenzo.muccioli@ausl.bologna.it (L.M.)

**Keywords:** seizure cluster, acute repetitive seizures, epilepsy, status epilepticus, ASAP, REST, ACT

## Abstract

Seizure clusters (SCs) are an acute and transient increase in seizure frequency relative to an individual patient’s baseline and are associated with an increased risk of injury, morbidity, and potentially mortality if not promptly and adequately treated. Despite their clinical importance, the management of SCs remains highly heterogeneous, primarily due to the absence of a universally accepted definition, which is determined also by the wide variability in seizure semiology and baseline individual burden;, as well as by differences in care settings. Outpatient treatment relies largely on caregivers’ ability to recognize SCs and administer rescue medication, whereas inpatient management may also involve invasive routes of administration. We conducted a literature review identifying 32 original articles addressing the treatment of SCs. The analysis focused on definitions, efficacy outcomes, and adverse events across three clinical scenarios: outpatient, Emergency Department (EDs) and Epilepsy Monitoring Units. The results show that in the outpatient setting, the available evidence suggests that diazepam nasal spray (DZP-NS), midazolam nasal spray (MDZ-NS), and oral lorazepam (LZP) solution may demonstrate comparable efficacy and safety. However, comparisons are limited by heterogeneity in studies’ designs, patient populations and outcome definitions, as well as by the absence of head-to-head trials. Moreover, geographic differences in drug availability (e.g., USA vs. Europe) limit the development of universally applicable treatment protocols. Consequently, the off-label use of oral benzodiazepines, including clobazam, clonazepam, and lorazepam, remains common when oral therapy is feasible, despite limited evidence. The implementation of a patient-specific Acute Seizure Action Plan (ASAP) incorporating an individualized SC definition is recommended. In contrast, inpatient management shows greater consensus, largely reflecting first-line treatment paradigms for status epilepticus. These include prompt intravenous benzodiazepine administration, followed by the intravenous loading of antiseizure medications such as brivaracetam or lacosamide in cases of seizure recurrence. In ED settings, “empirical” definitions of SCs (i.e., more than three seizures within 24 h) may facilitate timely intervention.

## 1. Introduction

A seizure cluster (SC), also referred to as acute repetitive seizures (ARS), is generally defined as a sudden increase in seizure frequency compared with an individual patient’s baseline, with recovery of the usual neurological status during the interictal period. Despite this general definition, and although SCs are frequently encountered in both outpatient and acute care settings, standardized diagnostic criteria are lacking.

SCs represent a substantial clinical and societal burden. For people with epilepsy (PWE) and their caregivers, their occurrence is often associated with significant psychological distress, anxiety, and a reduced sense of control over the disease. These factors negatively affect quality of life, daily functioning and productivity [1]. In particular, epidemiological data indicate that PWE with a history of SC are at significantly higher risk of developing status epilepticus (SE) than those without such a history (44% vs. 13%) [2]. Moreover, another interesting study underlies a strong association between SCs and a previous history of convulsive SE [3]. This association has also raised concerns about potential increase in mortality and the risk of sudden unexpected death in epilepsy (SUDEP) [4,5,6].

A history of SCs has been significantly associated with seizure-related hospitalization, independently of convulsive SE [3], whereas the use of rescue medication was significantly associated with fewer injuries and Emergency Department visits [7].

Several risk factors for SC have been consistently identified. These include drug-resistant epilepsy, a prior history of SE, and high baseline seizure frequency [8,9]. Additional factors—such as sleep deprivation, concomitant systemic disease, medication non-adherence, and hormonal influences—have also been implicated; however, the supporting evidence remains inconsistent [10]. The association between SC occurrence and the localization of the seizure onset zone in focal epilepsies is still unclear, as the literature reports conflicting or inconclusive findings [3,10,11].

The reported prevalence of SC varies widely, ranging from approximately 3% among PWE in large population-based studies to 46% in tertiary epilepsy centres [7,11,12,13,14]. This variability reflects differences in studies’ designs, patient populations, and clinical settings, as well as heterogeneity in the operational definitions of SC. A major challenge in both research and clinical practice is the absence of a universally accepted definition. So-called “empirical” or “clinical” criteria differ substantially across studies with regard to the number of seizures required to define a cluster and the time interval considered [9,10,13]. The commonly adopted empirical/clinical criteria include the following:

- Two or more seizures within 24 h;
- Two or more seizures within 6 h [15,16];
- Two to four seizures within 48 h;
- Two generalized tonic–clonic seizures or three focal seizures with impaired awareness within 4 h [9,10].

Alternatively, “statistical definitions” are based on deviations from an individual patient’s baseline seizure frequency [10].

A recent expert consensus on the outpatient management of SCs and prolonged seizures proposed defining SCs as an “abnormal increase in seizure frequency compared with the individual patient’s usual seizure pattern,” explicitly rejecting time-based definitions [17]. Clinicians are therefore encouraged to develop personalized treatment strategies, commonly referred to as Acute Seizure Action Plans (ASAPs) [18,19,20,21,22]. An ASAP is a written document developed in collaboration with the patient and caregivers. It describes the individual seizure pattern, specifies situations requiring rescue treatment and clarifies when emergency medical assistance should be sought [23]. In addition to improving clinical management, ASAPs may provide significant economic benefits [7].

The consensus also distinguished two therapeutic strategies. The first, termed ACT (Acute Cluster Treatment), aims to prevent additional seizures within a cluster. The second, termed REST (Rapid and Early Seizure Termination), is intended to stop an ongoing seizure. This distinction enables the categorization of medications according to their time to peak plasma concentration and duration of action, and supports the selection of the most appropriate route of administration for specific clinical scenarios. REST medications should exert their effect within approximately 2 min to achieve seizure termination [17].

Benzodiazepines (BZDs) remain the cornerstone of acute treatment, because of their rapid antiseizure activity and well-established efficacy. However, their pharmacokinetic profiles differ according to the specific molecule and formulation. Some BZDs (e.g., clobazam and clonazepam) are commonly used for the treatment of SCs despite lacking a specific regulatory indication. The availability of benzodiazepines and their routes of administration vary considerably across regions and healthcare systems (see Table 1). Consequently, the real-world management of SCs is highly heterogeneous. This variability is further driven by the abovementioned absence of a standardized definition. Additional factors, including caregiver education, patient comorbidities, disease severity, and access to healthcare services, also contribute to differences in treatment approaches.

In hospital settings, including Emergency Departments (ED) and Epilepsy Monitoring Units (EMU), treatment decisions are also influenced by the availability of intravenous access, monitoring capabilities, and local institutional protocols. The absence of standardized approaches may delay effective intervention and increase the risk of progression to SE.

In light of these challenges, a comprehensive and critical synthesis of the available evidence is essential to guide clinical decision-making. This review analyzes the current literature on the treatment of SCs in adult patients, with particular emphasis on pharmacological rescue therapies. Specific attention is given to differences between outpatient and inpatient settings, including EDs and EMUs, in order to address setting-specific issues related to drug availability, feasibility, and clinical priorities.

## 2. Methods

### 2.1. Search Strategy and Eligibility Criteria

A PubMed search was conducted in April 2025 using the following search strings: “(acute repetitive seizures [Title/Abstract]) NOT (veterinary)” and “(seizure cluster [Title/Abstract]) NOT (veterinary)”. The aim was to identify original studies reporting treatment options for SCs. Only articles published in English from 2005 onward were included to capture contemporary treatment strategies. The following exclusion criteria were applied:

- Review articles and other non-original contributions;
- Clinical pharmacology or preclinical studies;
- Studies exclusively involving pediatric populations, as the present review focuses on adult patients. Studies including both adults and pediatric patients were considered eligible; in such cases, the mean age of the overall study population was reported;
- Studies investigating therapies no longer available;
- Studies in which status epilepticus and SCs were analyzed as a single combined group.

### 2.2. Article Selection

The articles’ eligibility was assessed by a single reviewer (GB) through a two-step process: (1) the screening of the title and abstract and (2) full-text evaluation. Studies that clearly did not meet the inclusion criteria or clearly fulfilled any exclusion criteria were removed during the initial screening. The remaining articles underwent full-text review. The reasons for exclusion were documented and independently verified by a second reviewer (EP) to ensure accuracy. The reference lists of included studies were also examined to identify additional relevant publications. The final set of studies included in the analysis was reviewed and approved by all authors.

### 2.3. Data Analysis

Each study was assessed according to the following variables: study design, sample size, sex distribution, age, clinical setting (outpatient, Emergency Department, or Epilepsy Monitoring Unit), definition of SCs, seizure type, the inclusion of SE or other seizure emergencies, etiology, the inclusion of patients in whom SCs represented the first manifestation of epilepsy, baseline seizure frequency, SC treatment strategy, the number and type of antiseizure medications (ASMs), hospitalization, and diagnostic investigations.

Efficacy outcomes included all reported measures, such as time to drug administration, time to seizure cessation, seizure recurrence at 6, 12, and 24 h, need for a second dose within 6, 12, or 24 h, progression to SE, hospitalization, and drug-related adverse events (AEs).

## 3. Results

### 3.1. Study Selection

The PubMed search identified 207 articles. After removing 28 duplicates, 179 abstracts were screened. Of these, 68 were excluded as they did not meet the inclusion criteria, and 83 were excluded based on the predefined exclusion criteria: 36 were reviews or non-original articles, 24 were clinical pharmacology or preclinical studies, 18 involved pediatric-only populations, 3 investigated therapies no longer available, and 2 analyzed SE and SCs together.

**Figure 1:**
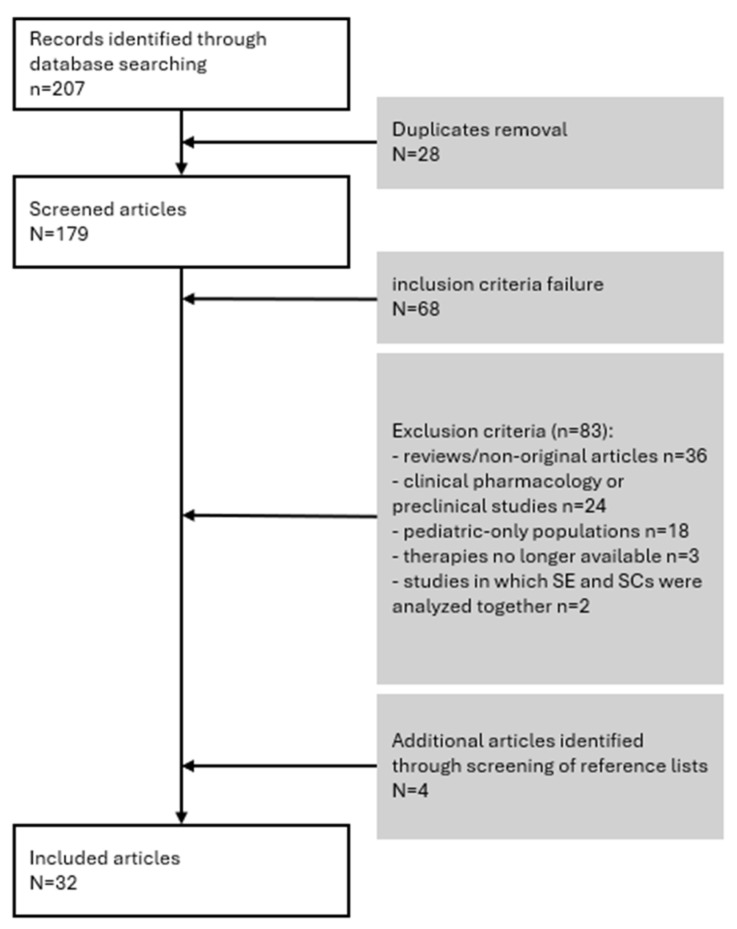
Studies’ selection: flow diagram.

The screening of reference lists identified four additional eligible publications [17,28,29,30]. Following the full-text review, a total of 32 articles were included in the analysis. Figure 1 illustrates the studies selection process.

### 3.2. Outpatient Management

Of the 32 selected studies, 22 (68.8%) focused on the outpatient setting. In these studies, medications were administered primarily by caregivers, and less frequently were self-administered by patients. All the studies evaluated BZDs, and diazepam (DZP) was evaluated in 15 studies and midazolam (MDZ) in 5 studies. In 19 of the 22 studies, BZDs were delivered via the intranasal (IN) route. The studies’ characteristics —including the design, sample size, and SC definition—are summarized in Table 2, organized by treatment strategy. Only one study was a double-blind randomized controlled trial (RCT) whereas the majority of evidence was derived from open-label trials or retrospective studies.

Four medications were prescribed in outpatient studies: MDZ-NS (nasal spray), DZP-NS, lorarepam (LZP) oral solution, and DZP rectal gel.

- MDZ-NS was administered at a fixed 5 mg dose, regardless of age or weight, for episodes, lasting ≥ 10 min, of ≥2 seizures (focal or generalized) within 6 h. A second 5 mg dose was allowed 10 min to 6 h later [15,16,31,32,33].
- DZP-NS was administered at 5–20 mg, depending on age and weight. A second dose was permitted 4–24 h later. Indication was broadly defined (“seizures that might require benzodiazepine intervention”) [28].
- LZP oral solution was prescribed at 0.5–2 mg (65% of the cohort received 1 mg; 29% 2 mg) for two or more seizures in 24 h (73% of patients) or prolonged seizures > 5 min (21%) [34].
- DZP rectal gel was evaluated in a single-centre retrospective study of 50 patients (mean age 34.7 years) for SCs (>2 seizures) or prolonged seizures. The doses ranged from 10 to 20 mg, adjusted according to weight [35].

**Table 2:** Characteristics of included outpatient studies, including study design, evaluated medication, sample size, and SC definition.

Treatment Strategy	Study	Study Design	N. Patients (% of Adults, Mean Age)	N. Treated SCs	SC Definition	Seizure Type
MDZ-NS (5 mg, ±second 5 mg dose)	Wheless et al. [16] Meng et al. [32] ^1^ Detyniecki et al. [31] ^1^	Open-label extension trial (single arm)	161 (95%; 32.9 years); 49.7% females	1998 *: 1291 with single dose; 797 with a second dose	≥2 seizures/6 h	focal or generalized
	Detyniecki et al. [15]	Phase 3 RCT double blind (vs. placebo 2:1)	67 in placebo, 133 in MDZ (96.3%; 34.0 years in MDZ), 49–55% and females	NA	≥2 seizures/6 h	focal or generalized
DZP-NS (5, 10, 15 or 20 mg depending on age and weight, ±second dose)	Wheless et al. [28] ^2^	Phase 3, open-label, repeat-dose safety study	163 * (NA, 23.1 years); 54.6% females	3853 *	NA	NA
	Liow et al. [36], Misra et al. [37]	Post hoc cohort analysis ^3^	NS [36] 151 [37]	485	≥2/24 h	NA
	Jarrar et al. [38]	Post hoc cohort analysis ^3^	NA	727	Prolonged seizures	NA
DZP-NS (5–20 mg according to age and weight) and MDZ-NS (5 mg)	Li et al. [33]	Retrospective	39 in DZP-NS, 38 in MDZ-NS (NA, median 25 and 27 years for DZP and MDZ respectively), and 46–58% females	NA	NA	NA
LZP oral solution 0.5–2 mg/dose (65% 1 mg, 29% 2 mg)	Lelis et al. [34]	Retrospective, single centre	48 (100%; 37.8 years); 40% females. 32 sublingual, 11 buccal, and 5 oral administration.	NA	Individual definition: ≥2/10 min–24 h or prolonged seizures	NA
BZD real world use	Chiang et al. [39]	Retrospective from Seizure Tracker database	220 (NA, 14.4 years); 52.7% females	10,889 rescue medication administrations	≥2/4 h or ≥2/6 h or ≥2/24 h	Focal impaired awareness seizures and bilateral tonic–clonic seizures
DZP rectal gel	Fakhoury et al. [35]	Retrospective, single centre	50 (100%; 34.7 years); 52% females	NA	NA	NA

NA = not available. ^1^ Post hoc analysis of the study by Wheless et al. [16]. ^2^ The following articles are interim analysis or post hoc analysis of this study: Miller et al. [40], Cascino et al. [41], Segal et al. [42], Wheless et al. [43], Misra et al. [44], Cramer et al. [45], Penovich et al. [46], Sperling et al. [47], Misra et al. [48], and Peters et al. [49]. ^3^ Post hoc cohort analysis of the above-mentioned trial by Wheless et al. [28]. * Post hoc analysis may have had a smaller sample.

Males and females were generally equally represented (detailed in Table 2). Ages ranged from 6 to 75 years, with younger patients included in DZP studies [28,33], whereas the LZP cohort comprised only adults [34].

Over 50% of patients had drug-resistant epilepsy. The baseline seizure frequency was reported in only one study [34]. MDZ-NS trials enrolled patients with high SC frequency (mean 7.97 episodes/year) [15,16]. Etiology was often unspecified, although the 40–66% of patients had encephalopathy or were dependent in daily activities [28,33,34].

The seizure types varied across studies. Tonic–clonic seizures in MDZ-NS and DZP-NS trials ranged between 40 and 70%. LZP oral solution was used to target mainly focal seizures with impaired awareness [34]. Notably, 6% of patients used LZP to prevent seizures following auras [34]. DZP-NS was administered “at the first sign of a seizure” by 8.8% of caregivers and 48% of self-administering patients; only 24% of self-administrations targeted SCs [46]. The reasons for these types of prescriptions were not specified. Data on SC precipitating factors were not available.

Efficacy outcomes were variably defined. Seizure freedom at 24 h ranged from 62.7 in a double-blind RCT (with a statistically significant difference from placebo) [15] to 63.1% in an open-label trial [16] with MDZ-NS treatment. Seizure freedom at 24 h reached 66% with LZP oral solution, and was higher (78%) when administered sublingually [34]. Data on seizure recurrence with DZP-NS were unavailable; 12.6% of patients required a second dose within 24 h [28,42,49]. No differences were observed in the number of second doses of DZP-NS in the case of chronic use of BZDs or concomitant cannabidiol use [42,49].

The return to baseline function occurred within 1 h in ~45% of MDZ-NS patients in an open-label study [31] and in 60% of DZP-NS patients [46]. The progression to SE was rare: 0–1.2% with MDZ-NS [15,16] and 4.3% with DZP-NS in another study [28].

No data were available regarding hospitalization reduction. Two studies reported that effective SC treatment may prevent subsequent clusters by prolonging the inter-cluster interval [37,39].

Overall AEs were reported in 18.4% of DZP-NS patients [28]. For MDZ-NS, AE rates ranged from 26.4% (versus 23.1% in the placebo group) [15] to 31.7% in an open-label single-arm study [16]. The overall safety profile was comparable across medications. Acute Central Respiratory Depression (ACRD) occurred in 0.7% of MDZ-NS patients during the test phase [15]. No serious drug-related AEs were reported with DZP-NS or LZP [28,34].

The efficacy outcomes and AEs reported in the selected outpatient studies are summarized in Table 3. Heterogeneity in the outcomes—including seizure cessation, seizure recurrence at various time points, the use of a second dose, and the return to full baseline functionality—is evident.

### 3.3. In-Hospital Management

Nine studies (28.1%) evaluated in-hospital SC management, including five studies conducted in the ED [50,51,52,53,54] and four in EMU [30,55,56,57].

The medications assessed included intranasal midazolam (IN-MDZ, referring to the intranasal atomization of the intramuscular MDZ formulation, and MDZ-NS) [30,55,57], intravenous LZP [56,57], and intravenous non-benzodiazepine ASMs—brivaracetam, lacosamide, valproic acid, and phenobarbital [50,51,52,53,56].

#### 3.3.1. Emergency Department (ED)

The studies’ characteristics—including the design, sample size, and SC definition—are summarized in Table 4. Only one study was an open-label RCT, whereas most of the evidence comes from retrospective or ambispective observational studies.

Within the selected time frame, all identified studies evaluated ASMs as add-on therapy after benzodiazepine failure or as first-line treatment in patients with contraindications to benzodiazepines.

- Intravenous brivaracetam (IV-BRV) was administered for the treatment of ≥2 seizures occurring within 6–24 h (depending on the study), considered infrequent for the patient [50,51]. A loading dose of ≥1.82 mg/kg was used, based on previous studies investigating IV-BRV for SE, followed by the maintenance therapy of 100–200 mg/day in 74% of patients [50]. IV-BRV was used as first-line therapy in 17–41.8% of cases and as second-line therapy in 35.8–49% [50,51].
- Intravenous lacosamide (IV-LCM) was added to standard ASMs for the treatment of ≥2 seizures occurring within 1 h, with the complete recovery to baseline between events. This presentation was considered atypical for the individual patient. The median loading dose was 200 mg [52].
- Intravenous valproic acid (IV-VPA, 30 mg/kg) and intravenous phenytoin (IV-PHT; 18 mg/kg) were evaluated as first-line therapy (without prior BZD administration) in patients experiencing ≥2 seizures over 5–6 h. These events differed from the patient’s usual seizure pattern in both frequency and severity. No maintenance doses were administered [53].

Males and females were generally equally represented, except in the IV-VPA and IV-PHT groups, which included a higher proportion of male patients [53]. The mean age of patients presenting to the ED for SCs ranged from 51 to 62.3 years [50,51,52,53], with higher mean ages reported in studies evaluating IV-BRV [50,51].

A total of 42.9–76.0% of patients had pre-existing epilepsy [50,51,52], whereas in the remaining cases, the SC represented the first epileptic manifestation. The most common etiology was structural [50,51,52], followed by unknown causes. Prior seizure frequency in PWE was not reported, nor was the proportion of drug-resistant cases. However, in one IV-BRV study, the mean number of concomitant ASMs was 2.3 [51].

ASM withdrawal, treatment non-adherence, or subtherapeutic drug levels accounted for 9.5–16.2% of cases across different subgroups [50,52,53]. Other frequent provoking factors included acute systemic illness (e.g., fever) and sleep deprivation. Nevertheless, most cases were classified as unprovoked [50]. Most treated seizures were focal in the IV-BRV [50,51] and IV-LCM [52] studies. In contrast, in the IV-VPA/IV-PHT group, the majority of patients were treated for tonic–clonic seizures [53].

Direct comparisons of efficacy are not possible because the outcome definitions varied across studies. The reported outcomes are summarized in Table 5. Seizure freedom within 24 h was reported in two studies: 58% with IV-BRV [50] and 67% with IV-LCM (83% when used as first-line therapy) [52]. Following IV-BRV administration, seizure freedom was significantly higher in PWE than in patients without a prior history of epilepsy. Moreover, SCs provoked by systemic factors were associated with a lower probability of seizure freedom and a higher risk of progression to SE in PWE [50].

The efficacy of IV-BRV and IV-LCM was not influenced by the seizure type in two studies [50,51,52]. Favourable outcomes were associated with a shorter time to treatment and earlier drug administration (first or second line) for both IV-BRV [50,51] and IV-LCM [52]. Rapid IV-BRV infusion (1–5 min) was also associated with improved outcomes [51]. No significant differences were observed between higher (>200 mg) and lower (<200 mg) IV-LCM loading doses [52].

Clinical response was confirmed electrographically in all patients treated with IV-LCM [52] and in at least 58% of those treated with IV-BRV [50,51,52]. In the study evaluating IV-VPA and IV-PHT [53], EEG was performed only when non-convulsive SE was suspected. The progression from SC to SE (17% of patients) was reported only in IV-BRV studies [50], as was subsequent hospitalization (92.9% of patients), with a mean length of stay of 15 days [51].

AEs were reported in fewer than 15% of the IV-BRV-treated patients in two studies [50,51,52] and in 16% of the IV-LCM-treated patients in another study [52]. The most common AEs included somnolence/drowsiness, dizziness, nausea, vomiting, fatigue and blurred vision. No serious AEs were reported with IV-BRV or IV-LCM. The efficacy outcomes and AEs from the ED studies are summarized in Table 5. As noted above, there is considerable variability in outcome definitions across studies.

#### 3.3.2. Epilepsy Monitoring Unit (EMU)

In the EMU setting, three of four studies evaluated benzodiazepine efficacy (MDZ-NS [55], IN-MDZ [30,57], or IV-LZP [57]), whereas one study compared the efficacy and safety of IV-BRV versus IV-LZP [56].

The studies’ characteristics, including their design, sample size, and SC definition, are summarized in Table 6. Only one study was a double-blind RCT; the remaining evidence derives from open-label or retrospective/ambispective observational studies.

As-needed therapy for increased seizure frequency, including SCs, was prescribed under the following conditions: ≥2 seizures within 6 h, ≥3 seizures within 24 h, or any generalized tonic–clonic seizure requiring treatment at the clinician’s discretion [55,56,57]. The main exclusion criteria were chronic benzodiazepine use (>3 times per week) in the MDZ-NS study [55] and recent (<28 days) initiation of BRV or use of chronic BZDs in the IV-BRV study [56].

Males and females were generally equally represented. The mean age of patients in the EMU ranged from 32.7 to 43.9 years [30,55,56,57].

When reported, most patients treated with MDZ, IV-LZP or IV-BRV were drug resistant, as expected in this population [42]. The baseline (out-of-cluster) seizure frequency was not reported. No data were available on seizure etiology. When specified, tonic–clonic seizures occurred in 60–93% of patients across the studies [30,55,56,57].

Efficacy was defined as the absence of seizure recurrence within 12 h: 20% with IV-BRV and 40% with IV-LZP in one study [56], and 42.6% with IN-MDZ in another [30]. In a double-blind RCT, seizure freedom at 6 h was achieved in 54.8% of patients treated with MDZ-NS, with no significant difference compared with placebo [55]. EEG monitoring, confirming electrographic seizure cessation, was performed in all patients [30,56,57].

AEs occurred in 42–60.9% of patients receiving MDZ-NS or IN-MDZ [55,57], 25–55.6% of those treated with IV-LZP [56,57], and in 13.0% of patients treated with IV-BRV. Nasal discomfort and throat irritation were the most common with MDZ-NS/IN-MDZ, whereas phlebitis or drug extravasation occurred in 7.4% of IV-LZP treated patients [57]. No cases of respiratory depression or other severe AEs were reported with any treatment. Progression to SE occurred in 0–4.3% of MDZ-treated patients [30,55,57] and in 0–14.8% of patients treated with IV-LZP studies [56,57], whereas no progression to SE was reported in the IV-BRV study [56]. No data were available regarding prolonged hospitalization due to SCs or increased seizure activity in the EMU.

The efficacy outcomes and AEs reported in EMU studies are summarized in Table 7. The variability in the definition of efficacy outcomes is noted.

## 4. Discussion

SCs represent a transient increase in seizure frequency. Currently, no universally accepted definition exists, and the literature is affected by heterogeneous criteria (summarized in the “SC definition” columns of Table 2, Table 4 and Table 6). “Clinical”/“empirical” definitions—based on the number of seizures within a specific time frame (e.g., ≥2 seizures within 24 h)—are convenient and easily applied in ED settings, where detailed knowledge of a patient’s seizure history may be lacking. However, in patients with a high baseline seizure frequency, there is a risk of overestimating the diagnosis of SC. To address this limitation, some studies have proposed a “statistical definition”, based on deviations from a patient’s baseline seizure frequency. For example, an increase by a factor of 3–4 over a 3-day period has been suggested as a valid criterion [10]. The clinical setting also critically influences the choice of antiseizure medication and the urgency of intervention. Patients presenting to the ED with recurrent seizures over the preceding hours typically require rapid treatment to terminate the cluster and reduce the risk of progression to SE. In contrast, patients experiencing predictable seizure exacerbations, such as during the menstrual cycle, may require preventive therapy over subsequent hours, with a different tolerance for side effects and acceptable seizure frequency.

Clinicians further emphasize the need to differentiate SC definitions based on patients’ characteristics and seizure types (focal vs. generalized, and with or without impaired awareness), which further limits the generalizability of a standardized approach. Additionally, the availability of medications across settings and differences in drug formulations between countries (e.g., USA vs. Europe) reduce the feasibility of developing universally applicable treatment algorithms or protocols (see Table 1).

This literature review aimed to analyze current evidence to guide medication prescription. The limitations of this study include that a formal bias assessment of the included studies was not performed. High-quality evidence—such as double-blind randomized controlled trials and head-to-head comparisons—remains scarce. Moreover, SCs and efficacy outcomes have been variably defined across different studies, in both outpatient and inpatient settings, which hampers comparison among different medications.

For outpatients, a recent expert consensus has recommended the use of an individualized SC definition for management [17]. Clinicians should develop ASAPs (Acute Seizure Action Plans) and prescribe rescue treatment following ACT or REST purpose (Table 8). Under this framework, the consensus recommends offering ACT prophylactically to all patients with a history of SCs to prevent further episodes, while REST should be prescribed to patients with a history of myoclonic or absence seizures known to progress to tonic–clonic seizures, those with prolonged seizures or SE, or patients with focal seizures without impaired awareness that evolve into seizures with impaired awareness or bilateral tonic–clonic seizures [17].

We concur that for outpatients an ASAP should be developed [18] collaboratively with the patient and caregiver for all individuals at risk of SCs or prolonged seizures. This plan should be tailored to the patient’s characteristics, including their independence in activities of daily living, occupational status, seizure type, and cluster duration.

Our literature review suggests that DZP-NS, MDZ-NS and LZP oral solution may exhibit a similar efficacy and safety profile, despite several limitations:

- Efficacy was defined using different criteria across studies;
- Studies’ designs varies considerably;
- No head-to-head comparisons are available.

Specifically, DZP-NS has not been evaluated in randomized controlled trials; available data derive from an open-label safety study and its post hoc analysis. MDZ-NS was assessed against placebo and, subsequently, in an open-label extension trial. The LZP data originates from a single-centre retrospective study. The discrepancy in second dose use (31.3–37.3% at 6 h with MDZ-NS vs. 12.6% with DZP-NS) may reflect the broader use of DZP-NS beyond SCs. However, the lack of availability of MDZ-NS and DZP-NS in some countries (see Table 1) limits their clinical use. Consequently, the off-label use of oral BZDs (i.e., clobazam and clonazepam, as well as the above-mentioned lorazepam) still remains common when oral therapy is feasible, despite limited supporting evidence.

Interestingly, cluster prevention may exert a long-term benefits by increasing the interval to the next cluster, suggesting that, at least in some patients, a self-perpetuating mechanism underlies SC recurrence [37,39]. The safety profile showed overall mild AEs.

Regarding SC management in EDs, the literature indicates substantial heterogeneity. In a significant proportion of cases, SCs represent the first epileptic manifestation [50,51,52]. Currently, no specific guidelines exist for managing this neurological emergency. Based on our review, there is a general consensus supporting the application of the same first-line treatment paradigm used for SE: initial IV benzodiazepine administration, followed—if seizures recur—by the IV loading of ASMs such as brivaracetam [50,51] or lacosamide [52]. Existing studies on intravenous ASMs raise the question of whether tolerability and the route of administration outweigh therapeutic indication. This conceptual limitation has also been noted in prior observational studies of SE and has been hypothesized as a factor contributing to worse prognostic outcomes [58]. While some findings suggest a potential advantage of brivaracetam over benzodiazepines in terms of reducing the risk of progression to SE, these observations require further investigation, particularly in patient populations with diverse seizure aetiologies. Prospective studies comparing the efficacy of rapid-acting benzodiazepines—potentially including intranasal formulations—versus ASMs in SC management are warranted.

The EMU setting presents distinct challenges. The rapid tapering of ASMs to facilitate seizure recording while minimizing hospitalization duration increases the risk of cluster seizures. Shih et al. (2025) surveyed over 20 experts from a dedicated Epilepsy Education Council and reported significant variability in cluster definitions, ranging from two seizures in 1 h to three seizures within 24 h [29]. This variability reflects both baseline patient characteristics and seizure semiology, with tonic–clonic seizures occurring more frequently during rapid medication tapering. According to the International League Against Epilepsy’s clinical practice guidelines, a fast taper (30–50%) or slow taper (15–30%) is considered safe for patients without a history of SE or frequent daily seizures during EMU hospitalization [59]. Rescue medications are typically predetermined by the supervising neurologist. Most experts reported preferring IV-LZP if available; otherwise, intranasal MDZ is used [29]. These choices largely depend on drug availability in different countries. In Europe, for example, buccal midazolam is commonly used both at home and in the hospital. Often, reloading the patient’s previous therapy—sometimes intravenously—may suffice to terminate the cluster in this setting.

The treatment objectives and strategies identified in this review, across different clinical settings, are summarized in Table 8.

## 5. Conclusions

Seizure clusters (SCs) are defined as an abnormal increase in seizure frequency relative to an individual patient’s baseline. However, the lack of standardized temporal criteria contributes to substantial heterogeneity in clinical practice. Furthermore, the existing literature employs heterogeneous definitions, which complicates inter-study comparisons. Management strategies vary according to the clinical setting, drug properties, patients’ characteristics and clinicians’ experience.

A more streamlined, setting-oriented approach may improve care. In outpatient settings, insufficient patient and caregiver education and the underuse of rescue therapies remain major limitations. Individualized action plans should be prioritized to enable timely intervention, empowering patients or caregivers to terminate prolonged seizures (REST) or ongoing seizure clusters (ACT). Currently available medications include diazepam nasal spray, midazolam nasal spray, and, although lacking a specific indication for SCs, lorazepam oral solution. Direct comparisons of efficacy and safety are limited by variable outcome definitions. In the Emergency Department, SCs can be managed with prompt benzodiazepine administration and the correction of reversible precipitants, consistent with early SE protocols. During long-term video EEG monitoring, treatment decisions must balance diagnostic objectives with patient safety. Early, context-specific intervention and the routine use of individualized rescue protocols are essential to reduce complications and the risk of progression to status epilepticus.

The unification of SC and efficacy definitions is needed to improve comparability in future studies.

## Figures and Tables

**Table 1:** Benzodiazepines with specific regulatory approval or commonly used on an experience-based basis for the treatment of seizure clusters across different healthcare systems, including relevant pharmacokinetic parameters.

Medication	Approved by	Time to Peak Plasma Concentrations (Minutes)	Elimination Half-Life (Hours)
Rectal diazepam	FDA (1997), EMA ^1^	10–45	46
Buccal Midazolam	EMA (2011)	30	2.3
Midazolam nasal spray	FDA (2019) EMA (2022), however commercially unavailable in several EU countries	10–15	3.1–3.8
Diazepam Nasal Spray	FDA (2020)	60–90	49–56
Intramuscular Midazolam	No specific authorization for SC	30	3.9
Clobazam tablets/oral suspension	off-label use	60–120	19–60
Clonazepam oral drops/tablets	off-label use	60–240	30–40
Lorazepam oral solution/tablets	off-label use	1 h	12

^1^ FDA = Food and Drug Administration; EMA = European Medicines Agency. References: [24,25,26,27].

**Table 3:** Efficacy outcomes and AEs reported in the selected outpatient studies.

Treatment Strategy	Seizure Cessation	Seizure Recurrence at 6 h	Use of a Second Dose			Return to Full Baseline Functionality		Drug-Related AEs (tot)	Drug-Related Serious AEs	Evolution to SE
			At 6 h	At 12 h	At 24 h	<1 h	<24 h			
MDZ-NS [15,16,31,33]	52–87.6%	31–41.8%	36.9–37.3%	31.3–39.9%	NA	44.7–45.9%	72.4–74.0%	26.4–31.7%	0.7–1.2%	0–1.2%
									0.7% ACRD during test phase [15]	
DZP-NS [28,38,44,46]	63%	NA	NA	5.8% (224/3853)	12.6%	59.4% of patients	90.7%	18.4%	none [28]	4.3%,
										SC in 1.2%
Oral/sublingual/buccal LZP [34]	NA	NA	34%	NA	NA	NA	NA	NA	NA	NA
Rectal DZP [35]	NA	NA	90% ^1^	NA	NA	NA	NA	94%	NA	NA

^1^ “seizure not stopping”; the time is not specified. NA = not available. ACRD = Acute Central Respiratory Depression.

**Table 4:** Characteristics of the included studies regarding the Emergency Department setting (study design, evaluated medication, sample size, and SC definition).

Treatment Strategy	Study	Study Design	N. Patients (% of Adults, Mean Age)	SC Definition
IV-BRV (mean loading dose: 145.0 ± 66.2 mg in patients with urgent seizures)	Villanueva et al. [51]	retrospective multicentric study	69 of 156 * (98%; 57.7 years); 44.9% females	≥2/6 h
IV-BRV (loading dose of ≥1.82 mg/kg, maintenance dose of 100/150/200 mg/die according to clinical decision)	Orlandi et al. [50]	retrospective multicentric study	97 (100%; 62.3 years); 52% females	≥2/24 h ^1^
IV-LCM used as add-on to BZD, PHT, VPA and LEV (median loading dose 200 mg)	Garcés et al. [52]	retrospective multicentric study	43 of 98 * (100%; 53.0 years); 51.2% females	≥2 seizure/1 h
IV-VPA (30 mg/kg) vs. PHT (18 mg/kg), as first ASM (no prior-BZD)	Gilad et al. [53]	Randomized clinical trial; open label	47 of 74 *: 31 in IV-VPA, 16 in PHT (100%; 54.4 and 51 years in VPA and PHT respectively); % of females among patients treated for SC is not specified.	≥2/5–6 h
Use of a “seizure emergency code” strategy	Espinosa-Jovel et al. [54]	Observational, analytical, ambispective cohort study	336: 94 in the pre-seizure code period; 242 in the post-seizure code period. 43.5% were SCs (100%; median 37 years); 43.2% females	≥3/24 h

^1^ In patients who had not experienced seizures in last 21 days. * The sample also included patients treated for SE.

**Table 5:** Efficacy outcomes and AEs reported in the selected ED studies. Time to drug administration was not reported in any study. The use of a second dose at 12 h was also not reported.

Treatment Strategy	Seizure Recurrence			Use of a Second Dose		Time to Seizure Termination	Drug-Related AEs (tot)	Drug-Related Serious AEs	Evolution to SE
	At 6 h	At 12 h	At 24h	At 6h	At 24 h	Median			
IV-BRV [50,51]	28%	30.3–36%	42%	NA	9–23%	NA	6–14.7% *	0%	17%
IV-LCM [52]	NA	NA	32.6%	NA	16.2%	6.5 h	16.3%	NA	NA
IV-VPA [53]	NA	NA	NA	3.2% (within 20 min of the infusion)	NA	NA	None	None	NA
IV-PHT [53]	NA	NA	NA	6.3% (within 20 min of the infusion)	NA	NA	12%	2 patients	NA

* Considered overall (SE and other urgent seizures). NA = not available.

**Table 6:** Characteristics of the included studies regarding the Epilepsy Monitoring Unit setting (study design, evaluated medication, sample size, and SC definition).

Treatment Strategy	Study	Study Design	N. Patients (% of Adults, Mean Age)	N. Treated SCs	SC Definition
MDZ-NS (5 mg; single dose)	Spencer et al. [55]	Double-blind RCT (vs. placebo)	31 MDZ, 31 placebo (93.5%, 32.7–35.9 years), and 58.1–67.7% females	NA	≥2/6 h
IN-MDZ (3 mg; atomization of i.m. formulation) vs. IV-LZP (1 or 2 mg)	Owusu et al. [57]	Ambispective observational, and single centre	23 in MDZ-NS, 27 in IV-LZP (100%; 38.6–40.0 years), and 43.5–48.1% females	NA	≥2/6 h
IV-BRV 100 mg vs. IV-BRV 200 mg vs. IV-LZP (1–4 mg)	Szaflarski et al. [56]	Open-label RCT	16 IV-LZP, 15 IV-BRV 100 mg, and 15 IV-BRV 200 mg (100%; 40.2–43.9 years); females: 66.7% in LZP and 26.7–43.3% in BRV groups	NA	≥3/24 h or ≥2/6 h
IN-MDZ (5 mg, up to 10 mg)	Kay et al. [30]	Retrospective, observational, and single centre	75 (NA, 35.1 years); 44.9% females	110	“as needed”, by the attending physician

NA = not available.

**Table 7:** Efficacy outcomes and AEs reported in the selected EMU studies.

Treatment Strategy	Time to Administration	Seizure Recurrence			Use of a Second Dose			Time to Seizure Termination	Drug-Related AEs (tot)	Drug-Related Serious AEs	Evolution to SE	Prolonged Hospitalization
		6 h	12 h	4 h	6 h	12 h	24 h					
MDZ-NS/IN-MDZ [30,55,57]	2.17 min from EEG seizure onset; 2.03 min after clinical seizure onset	27–45.2%	42.6%	60.4%	NA	NA	30.4%	3.2 min	5.3–42–60.9%	None [55]/NA	0–4.3%	ICU transfer 0%
										No episodes of ACRD		
IV-LZP	NA	NA	40.0%	NA	26.7%	40.0%	29.6%	3.3 min	25–55.6%	6.3% (1 patient)	0–14.8%	ICU transfer 0%
[56,57]										No episodes of ACRD		
IV-BRV	NA	NA	20%	NA	6.70%	10%	NA	No patients seizing at the end of administration	13%	None	0%	92.9% admitted to hospital; mean length 15.0 days.
[56]												ICU transfer 17.9%

NA = not available, ACRD = Acute Central Respiratory Depression, and ICU = Intensive Care Unit.

**Table 8:** Treatment objectives and strategies identified in this review, across different clinical settings.

	Outpatient	Emergency Department	Epilepsy Monitoring Unit
**SC definition**	Included studies used variable, non-comparable definitions. According to recent consensus [17], individualized definition should be used.	Ranging from ≥2 seizure/1 h to ≥2 seizure/24 h	Ranging from ≥2/6 h to ≥3/24 h. However, often established at clinician’s discretion
**Main goal**	ACT (Acute Cluster Treatment) is aimed to prevent further seizures in a SC. REST (Rapid and Early Seizure Treatment) is aimed to interrupt an ongoing seizure [17].	Prevent further seizures in a SC and progression to SE	Prevent progression to SE
**Secondary goals**	Prevention of progression to SE. Prevention of progression to more severe seizure types. Reducing hospitalization risk. Reducing the time to return to baseline functionality of the patient and the burden on the caregiver [17].	Prevent hospitalization	Prevent prolonged hospitalization
**Medications with evidence from this review**	For ACT: MDZ-NS (5–10 mg); DZP-NS (5–20 mg); LZP oral solution (0.5–2 mg). REST drugs are currently anavailable; ideally should start acting within 2 min.	IV-BRV IV-LCM IV-VPA IV-PHT As first line or following IV-BZDs	MDZ-NS (5 mg) IN-MDZ (3–10 mg) IV-LZP (1–4 mg) IV-BRV (100 or 200 mg)
**Additional treatment strategies**	/	Identify and treat provoking factors	Reload chronic ASM therapy

## Data Availability

No new data were created.

## Abbreviations

The following abbreviations are used in this manuscript:

SC	Seizure Cluster
ARS	Acute Repetitive Seizures
PWE	People With Epilepsy
SE	Status Epilepticus
SUDEP	Sudden Unexpected Death in Epilepsy
ASAP	Acute Seizure Action Plans
ACT	Acute Cluster Treatment
REST	Rapid and Early Seizure Termination
BZDs	Benzodiazepines
FDA	Food and Drug Administration
EMA	European Medicines Agency
ED	Emergency Department
EMU	Epilepsy Monitoring Unit
ASMs	Antiseizure medications
AEs	Adverse Events
DZP	Diazepam
MDZ	Midazolam
IN	Intranasal
RCT	Randomized Controlled Trial
MDZ-NS	Midazolam Nasal Spray
DZP-NS	Diazepam Nasal Spray
LZP	Lorazepam
IN-MDZ	Intranasal Midazolam (refers to intranasal atomization of intramuscular midazolam)
IV-BRV	Intravenous Brivaracetam
IV-LCM	Intravenous Lacosamide
IV-VPA	Intravenous Valproic Acid
IV-PHT	Intravenous Phenobarbital
LEV	Levetiracetam
ACRD	Acute Central Respiratory Depression
ICU	Intensive Care Unit
